# Tangled in a Web: Management Type and Vegetation Shape the Occurrence of Web-Building Spiders in Protected Areas

**DOI:** 10.3390/insects13121129

**Published:** 2022-12-07

**Authors:** El Ellsworth, Yihan Li, Lenin D. Chari, Aidan Kron, Sydney Moyo

**Affiliations:** 1 Department of Biology and Program in Environmental Studies and Sciences, Rhodes College, Memphis, TN 38112, USA; 2 Department of Zoology and Entomology, Rhodes University, Makhanda 6140, South Africa

**Keywords:** invasive, tennessee, architecture, management, plants, IndVal

## Abstract

**Simple Summary:**

Spiders are among the most common predators in terrestrial ecosystems and play a crucial role in ecosystems. However, with changing environments, spiders are under pressure from pollution and habitat destruction. In this study, we collected spiders from five parks with different management histories in the greater Memphis, Tennessee area to explore the extent to which human oversight and management of natural areas, especially invasive plant management, influence spider occurrence. Our results showed that invasive plants might provide a valuable habitat for the humpbacked orb-weaver, which was predominantly found on invasive plant species. These findings may have implications for the management of invasive plants in parks and other protected areas.

**Abstract:**

Land management of parks and vegetation complexity can affect arthropod diversity and subsequently alter trophic interactions between predators and their prey. In this study, we examined spiders in five parks with varying management histories and intensities to determine whether certain spider species were associated with particular plants. We also determined whether web architecture influenced spider occurrence. Our results showed that humpbacked orb-weavers (*Eustala anastera*) were associated with an invasive plant, Chinese privet (*Ligustrum sinense*). This study revealed how invasive plants can potentially influence certain spider communities, as evidenced by this native spider species only occurring on invasive plants. Knowing more about spider populations—including species makeup and plants they populate—will give insights into how spider populations are dealing with various ecosystem changes. While we did not assess the effect of invasive plants on the behavior of spiders, it is possible that invasive species may not always be harmful to ecosystems; in the case of spiders, invasive plants may serve as a useful environment to live in. More studies are needed to ascertain whether invasive plants can have adverse effects on spider ecology in the long term.

## 1. Introduction

The role of biological invasions has been emphasized by many conservationists, with many of the findings showing that biological invasions can have striking ecological and socio-economic consequences [1,2,3]. Invasive alien plants are known to be one of the major drivers of global biodiversity declines [4,5]. In addition to potentially causing biodiversity declines, invasive plant eradication can be a costly endeavor for land managers [6].

At the epicenter of biological invasions are anthropogenic-related stressors. For instance, anthropogenic activities such as urbanization and globalization have contributed to the influx of invasive species in ecosystems globally, particularly after the Industrial Revolution and in the modern era [7]. Globally, 37% of all first records of invasive species were reported between 1970–2014 [7]; Europe alone had more invasive plant, mammal, and invertebrate introductions in 1975–2000 compared to any other time after the 16th century [8]. Such invasive species tend to displace endemic species through competition and can create noticeable changes in environments and ecosystem dynamics, particularly deleterious ones, which has greatly concerned scientists [9]. For instance, invasive species in Australia caused huge declines in native species populations [10]. Consequently, these invasions are of foremost importance and concern, as they disrupt the ecosystem balance maintained by native species. Plants in particular are of significant importance in ecosystems, as they provide many ecosystem services including food resources, hiding spots, and micro-habitats. As a result, when foreign plants are introduced, they may cause cascading effects that affect different levels of an ecosystem, including animal habitats, species distributions, and ecological roles. For example, native plants in Rwanda are important in preserving ideal physicochemical soil properties for soil-litter arthropods, supporting greater arthropod abundance compared to exotic plants [11].

Several researchers have studied the ways to mitigate the negative impacts of invasive plants through invasive species management (ISM), which costs billions of dollars annually [12]. However, it is worth noting that biological invasions are not straightforward issues. Invasive species management interacts with social, economic, and environmental factors, and if carried out improperly, it can cause unintended consequences [13]. One such effort was in a Hawaiian lowland wet forest where managers removed invasive plants over a decade to promote native biodiversity and return the forest to its pre-invaded state [14]. Subsequently, the removal of invasive plants unintentionally recruited even more invasive species [14]. Situations are further complicated in areas with well-established invasive species. For example, some research suggests that interdependency has emerged between local avian frugivore species and the invasive *Lonicera* species in Central Pennsylvania, USA, and direct removal may cause undesirable decrease in frugivore abundance [15]. Certain species have successfully adapted to environmental changes caused by invasive plants. For example, while invasive plants can have negative impacts on herbivores due to unpalatability, they provide greater structural complexity for various invertebrates [16,17,18]. Inherent biases might cause people, including scientists, to categorize invasive species and their impact on native ecosystems as singularly bad, despite demonstrations that natural invasions rarely caused notable ecological damage or reduced species richness [19,20,21]. As such, many scholars have suggested looking at introductions of invasive species as accidental experiments and quantifying their subsequent impacts in an unbiased way as to fully explore how ecosystem dynamics change when invasives establish themselves [22]. The aforementioned studies reveal the complex interactions between invasive plants and terrestrial organisms. To fully understand the extent of invasive species on environments, further research is needed on how invasives impact specific wildlife species in specific areas [23].

Invertebrates, specifically spiders, may serve as good biological indicators of overall ecosystem health for several reasons. First, with an annual prey kill of 400-800 million tons (compared to around 400 million tons of annual human meat and fish consumption), spider communities play important roles in the terrestrial ecosystem and ecosystem dynamics worldwide [24]. Second, because many spiders are habitat specialists that react to environmental changes and stress [25,26], they may be good model organisms to study habitat perturbations. Lastly, spider diversity is usually not related to plant species numbers [25,27,28] but rather to the structure (e.g., number of contact points) and microclimate of the habitat [29,30], which makes vegetation structure a good predictor of spider community occurrence. Because web-building spiders (e.g., the families Araneidae, Nephilidae, Tetragnathidae, Therididae, Uloboridae, Linyphiidae) often use plants as a substrate to spin their webs, their fitness and role in ecosystems can rely on plants [31,32]. This makes spiders a good model system to explore some of the impacts that invasive plants may have on terrestrial ecosystems.

To gain insights into the effects of human management of invasive plant species on local ecosystem, we examined how spiders, one of the most ubiquitous predators, interact with environments of varying plant distributions and ISM histories. To this end, we addressed the following guiding questions: (1) How do web characteristics and prey types relate to spider species occurrence? (2) Which parks and trees are predominantly associated with select species of spiders? (3) How is spider ecology potentially impacted by differing invasive plant species distributions?

## 2. Materials and Methods

### 2.1. Study Area and Site Descriptions

**Figure 1:**
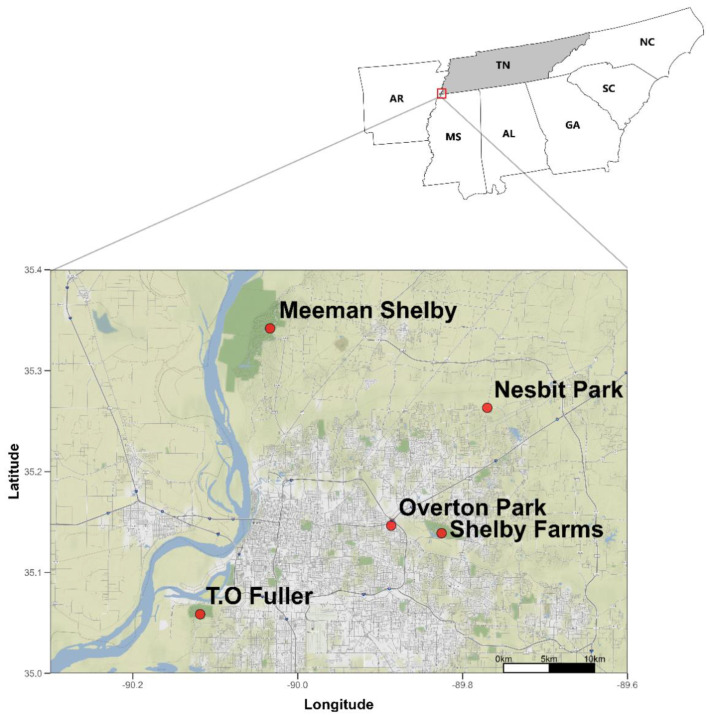
Map of Memphis showing five parks sampled during this study. Samples were collected in Memphis, Tennessee, USA. Maps and drawings were drawn using the ‘ggmap’ package in R [35].

Specimens were collected from five parks within the greater Memphis, Tennessee area (Figure 1). We selected these five parks because of the heterogeneity in their management histories (Table 1). It was assumed that different management practices would translate into differences in plant structure and communities. The tree species at these parks (Figure 1) have been adequately characterized based on the dominant vegetation (Laport, unpublished notes), making the endeavor in our study feasible. In addition, we selected sites within parks with previously characterized vegetation communities (Laport, unpublished notes). Climate, rainfall, and seasons are described elsewhere [33,34].

### 2.2. Sample Collections and Laboratory Processing

Spider collections were restricted to spiders found within webs in the field. In theory, this was an effort to predominantly collect specimens from web-building spider families (e.g., Araneidae, Tetragnathidae, Theridiidae and Linyphiidae) which utilize plants as a web anchor. Because a number of spiders rarely crawl on the ground and are restricted to their webs and the tree branches, active samplings via aerial hand collections were the most appropriate and conservative sampling method [39]. Active sampling is considerably less destructive to invertebrate microhabitats relative to methods such as beating and fogging that disturb or kill non-target invertebrates [40,41]. This was important to minimize our footprint on the environment during our study and preserve invertebrate biodiversity. Spiders were therefore collected using the hand-to-jar technique [42]. Samplings were conducted on a catch-per-unit-area basis to gain information on different habitats. Time and effort were standardized in each transect twice (beginning of the summer and end of the summer of 2022), and the same transects were used in subsequent sampling trips. In each season, at each site, intensive searches for web-building spiders were performed by two collectors within each transect. All collections took place between 06h00 and 12h00. Due to the difficulty in collecting spiders in out-of-reach heights, searches were restricted to a maximum vertical height of 2 m. Collections were conducted on climatically stable days (i.e., clear days without heat advisories or rainfall). A commercial hand-operated water sprayer (for ornamental plants) was used to increase the visibility of spider webs. After a spider was encountered, we measured the angle of the web in relation to the ground using a digital angle ruler. We measured web angles because web angles are known to affect rates of prey capture [43]. Similarly, web angles are correlated to wind conditions [44,45]. Moreover, webs are related to habitat with horizontal webs occurring in forest edges and riverine microhabitats [46,47]. We also recorded the number of contact points between web and plants (a proxy of vegetation structure/complexity) [48,49], a rough sketch of the web, plant species the web was constructed on, number of prey items within the web (proxy for successful predation), and GPS coordinates. Spiders were collected into 80 mL plastic vials, one per tube to prevent post-capture predation or cannibalism. After initial collection, specimens were labeled and frozen at −40 °C (to preserve coloration for ease of identification) until further analysis. Once frozen, specimens were sorted and identified by placing them on Petri dishes and then examining them under a dissecting microscope (AmScope, Irvine, CA, USA). Spiders were identified following Ubick et al. [50] and Bradley [51]. In instances where we could not adequately link spiders to species, we identified them by genera. Once specimens were identified and confirmed by a second researcher, ethanol was added to the vials for long-term preservation [51].

### 2.3. Data Analyses

The effects of vegetation (contact points), prey availability, and web orientation were investigated using a Canonical correspondence analysis (CCA) with abundance data. This technique was particularly appropriate to our data because it addresses the double-zero problem which characterizes community compositional data [52] and does not try to display all variation in the data but only the part that can be explained by the constraints considered [53]. Permutation tests (999 permutations) were run to assess model significance. The sum of the canonical eigenvalues was used as a measure of the variability in the response variables explained by predictors. Analyses were conducted in R using the “vegan” package [54].

We used indicator species analysis IndVal [55] to: (1) identify spider species associated with different parks and (2) identify spider species associated with particular tree species. We chose to use an IndVal analysis because this method identifies indicator species based on specificity (proportion of relative abundances of a taxon found in one park versus other parks) and fidelity (frequency of particular spiders in a particular park). Both parameters range from 0 to 1; specificity is 1 if a taxon is exclusively present in the target treatment, and fidelity is 1 when a taxon is present in all samples of the target treatment. IndVal for each taxon is the product of specificity and fidelity [56]. We performed the analysis at the genus level, used 999 random permutations, and considered spider taxa to be associated with particular parks or tree species if they were significant according to the IndVal permutation tests.

We performed all statistical analyses and graphical representations in R (version 4.1.3, Vienna, Austria) [57]

## 3. Results

### 3.1. Species Occurrence and Predictors of Their Occurrence (Question 1)

From the five parks sampled, we recorded 26 species from over 200 individual adults present on webs during the daytime. The most ubiquitous species was the orchard spider (*Leucauge venusta*) followed by basilica orb-weavers (*Mecynogea lemniscata*).

**Figure 2:**
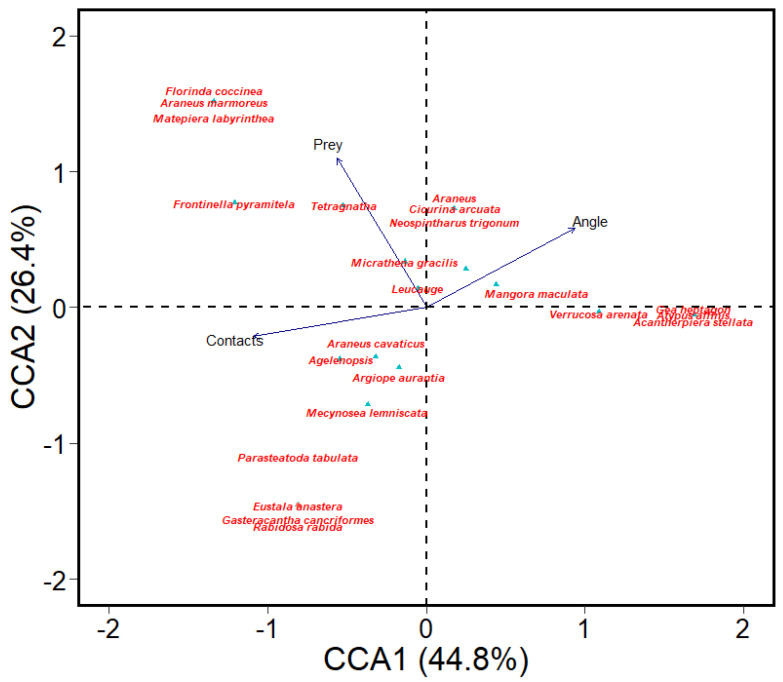
Canonical correspondence analysis (CCA) biplot showing the relationship between spiders and environmental variables (number of contact points (branches), prey abundance enumerated from webs and angle of webs). Samples were collected from parks in Memphis, TN in the summer months of 2022. Blue triangles denote species abundances.

CCA ordination of pooled samples (Figure 2) indicated that variation in spider occurrences was significantly related to prey (correlation = 0.51) and angle of webs (correlation = 0.73). Specifically, high abundances of *Tetragnatha* sp., *Micrathena gracilis*, and *Leucauge venusta* were positively related to prey availability. Web angle was positively correlated to *Mangora maculata* and negatively correlated to *Araneus cavaticus*, *Agelenopsis* sp., and *Argiope aurantia*. Sites with high abundances of spiders such as *Eustala anastera*, *Verrucosa arenata*, *Gea heptagon*, *Atypus affinis*, and *Acanthepeira stellata* were not related to any of the three variables measured (Figure 2). Contact points were not significantly related to spider occurrences.

### 3.2. Habitat Association and Indicator Species (Question 2)

IndVal analysis by habitat and tree species samples revealed the different species that are associated with certain habitats. For instance, Eustala anastera showed an association with Shelby Farms. It is worth noting that the greenlegged orb-weaver (Mangora maculata) was only found from samplings in Nesbit Park (Table 2). Considering the association between spiders and tree species (Table 3) revealed that deadwood was associated with non-web-building spiders (e.g., *Rabidosa rabida*), and web-building spiders (e.g., *Leucauge venusta*) were associated with deadwood in all parks, suggesting the importance of deadwood as a suitable habitat for spiders. Interestingly, *Cicurina arcuata* was only associated with tulip poplar trees. The invasive species Chinese privet (*Ligustrum sinense*) was associated with *Eustala anastera* across all sampled sites.

## 4. Discussion

We investigated the association of spiders and trees found in parks within a temperate deciduous forest biome. Our results revealed that species such as *Leucauge venusta* and *Mecynogea lemniscata* were the most abundant species recorded across all five parks. One key finding of this work is that the local invasive species Chinese privet is associated with *Eustala anastera*. These findings may have implications for the web-building spider *Eustala anastera* considering that many of these parks have goals to clear all invasives in their parks (Overton Park Conservancy, *personal communication*). Interestingly, other *Eustala* spider species are noted to have associations with particular plants. For instance, researchers in Panama found that *Eustala oblonga* and *Eustala illicita* spiders showed obligate preferences for acacia plants across all studied habitats [58]. Considering this preference for one plant type in many *Eustala* species [58,59], it is tenable that *Eustala anastera* spiders (such as the ones we sampled) may similarly form associations with privet in their environment. Our future work will incorporate greenhouse experiments with different plants to observe potential specificity of *Eustala anastera*.

Our results indicated that differences in plant distribution and spider micro-habitats across different parks (Table 2) are the likely causes of differences in web-building spider communities across these areas. These results confirm a previous study demonstrating that location and habitat characteristics, such as within-field location and landscape features, influence spider presence [60]. For example, spider site preference (tenure), an example of location and landscape features influencing spider presence, has been observed in some long-jawed spiders (*Tetragnatha elongata*), whereby many of member of this species will build webs very close to rivers and only at particular locations [61].

We found that many spider species were associated with particular plants (Table 3) in accordance with other researchers [31,62,63,64] The association between certain spiders with certain plants across different sites in our study indicates that invasive plant species might interact with specific local spider species in a complex way. Many studies focus on how invasive plants negatively impact herbivorous or native plants [65], and studies on the dynamics between invertebrates and invasive plants offer a more holistic view. Though invasive plants might outcompete native plant species, other native species within ecosystems, such as native invertebrates, can show abilities to adapt to new flora and even benefit from it. For example, researchers have noted the positive benefits that invasive plants can have on spider ecology [16,17]. At the same time, spiders are not the only invertebrates who benefit from the increase in vegetation cover: crickets, slugs, millipedes, and beetle populations are shown to have a positive relationship with vegetation cover too [66].

Our finding further supports the need for case-by-case analysis when resolving the question of invasive species control. In some situations, invasive species eradication has had great success in protecting endangered species [67], and in other situations invasive species can provide wildlife habitat and food resources for local individuals. In the case of Chinese privet, it was introduced to the US in the 1800s, allowing the species to fully expand geographically and co-evolve alongside native species through environmental changes. For invasions at such a large scale, it is time-consuming and economically costly to eradicate the species, and full removal might not return the ecosystem to what it was before the invasion [68]. This is not to say that there should be no control of invasive species or no preservation or protection of native species. In fact, there are certainly native species that do not benefit from invasive plants. For example, many native species in South Africa have been harmed due to the introduction of the invasive Australian *Acacia* causing significant declines in native species richness [69]. However, even with our best efforts, we cannot erase the contribution of globalization and humanity to speeding up the rate of invasive introductions. Scientists should be hesitant to outright list invasive species as automatically bad, and fully examine the impacts invasives have on ecosystems on a case-by-case, species-by-species basis, especially because certain native species have benefitted from invasive species. There is no guarantee that sudden removal of invasives would provide the best benefit to an ecosystem. Due to the benefits invasive plants provide invertebrates through additional surface-area coverage, other studies have suggested seeing whether its beneficial to partially manage invasives, just enough to preserve the benefits that invasives offer certain native species [17], an aspect encapsulated in functional eradication [70]. Thus, further research on how spiders and other invertebrates that inhabit certain invasive plants adjust to their removals is of interest.

Future directions include expanding on this study and consideration of other variables. For example, our study was limited to a specific geographical and temporal range, and as a result, we might not have found certain spider species in certain parks simply because we did not conduct long-term monitoring. Similarly, many spiders build webs at night, but our sampling took place during the daytime, so we might not have found certain spider species because our research had a diurnal spider bias. Having larger sample sizes, conducting more long-term research, conducting research during both night and day, or looking into species-specific analyses on how invasive species impact native species, for example, might give a more holistic and broader view on how invasives impact ecosystem dynamics and give more precise insights into populations in specific areas. Furthermore, expanding research to invertebrates and other relatively understudied species in comparison to mammals offers greater overall insight into how changing plant landscapes are impacting native ecosystems. In terms of spider ecology, future directions might include looking into how spiders in different regions utilize invasive plant species. Our study was limited to five local parks in Southwestern Tennessee; thus, making inferences for other parks in other regions must be conducted with caution. Further studies should investigate how plantation distribution affects prey abundance in answering the association between certain spider species with plant species.

## 5. Conclusions

In summary, we found that Chinese privet (*Ligustrum sinense*) is associated with the humpbacked orb-weaver (*Eustala anastera*). These findings may have implications for this spider species considering many of these parks have goals to clear all invasives in their parks. Our study makes an effort to contribute to spider research and conservation initiatives in that it provides baseline regional data that can be used to assess the effects of invasive plants in local protected areas. Future studies should consider sampling across larger spatial scales to determine whether the patterns documented here would be true for other ecosystems.

## Figures and Tables

**Table 1:** Site descriptions for samples collected in Memphis, TN, Summer 2022. Shelby Farms and Overton have more overall current funding, and consequently more management and human oversight, than Nesbit, Meem.an-Shelby, and T.O. Fuller [36,37,38].

Site	Description	Dominant Plants	Invasive
Shelby Farms Park	4500-acre public park that was opened for public recreation by the Shelby County government in the 1970s; is heavily managed, including forest trail upkeep.	Winged Elm Slippery Elm Privet	No No Yes
Overton Park Conservatory	184-acre public park opened in 2011 with heavy maintenance, including invasive species removals and trail upkeep.	Hornwort Pawpaw Box Elder Sweetgum	No No No No
Nesbit Park	333.75-acre park owned by cycling club company Stanking Creek Cycling open for free public use. No explicit plant management other than trail upkeep.	Jumpseed Pawpaw Winged Elm Sweetgum	No No No No
Meeman-Shelby State Park	13,469-acre state park initially built in the 1930s by the National Park Service; had land clearing and replanting efforts when built but currently has less spending management than Shelby Farms or Overton Parks.	Hophornbeam Beech Hophornbeam Sugar Maple	No No No No
T.O. Fuller State Park	1138-acre state park opened in 1938; has public recreation options but significantly lower revenues and spending than Shelby Farms and Overton Parks in recent years.	Pawpaw Privet Winged Elm Slippery Elm	No Yes No No

**Table 2:** IndVal analysis of species that are associated with specific parks based on the specimens we collected in the summer of 2022. Only significant (*p* < 0.05) habitat associations are presented. The significance of IndVal indices was assessed using 10,000 Monte Carlo permutations.

Park	Species	IndVal	*p*	Freq
Shelby Farms	*Eustala anastera*	1.00	0.013	3
Shelby Farms	*Parasteatoda tabulata*	1.00	0.01	3
Shelby Farms	*Gasteracantha cancriformes*	1.00	0.014	3
Shelby Farms	*Rabidosa rabida*	1.00	0.009	3
Shelby Farms	*Mecynogea lemniscata*	0.58	0.012	9
Shelby Farms	*Agelenopsis* sp.	0.53	0.012	12
Overton Park	*Cicurina arcuata*	1.00	0.012	3
Overton Park	*Araneid* sp.	1.00	0.013	3
Overton Park	*Neospintharus trigonum*	1.00	0.016	3
Overton Park	*Leucauge venusta*	0.36	0.014	15
Meeman-Shelby	*Florinda coccinea*	1.00	0.013	3
Meeman-Shelby	*Araneus marmoreus*	1.00	0.009	3
Meeman-Shelby	*Melpomene* sp.	1.00	0.011	3
Meeman-Shelby	*Metepiera labyrinthea*	1.00	0.022	3
Meeman-Shelby	*Frontinella pyramitela*	0.75	0.013	6
T.O Fuller	*Atypus affinis*	1.00	0.01	3
T.O Fuller	*Acantherpiera stellata*	1.00	0.009	3
T.O Fuller	*Gea heptagon*	1.00	0.015	3
T.O Fuller	*Verrucosa arenata*	0.74	0.017	12
Nesbit	*Mangora maculata*	0.50	0.015	15

**Table 3:** IndVal analysis of species that were associated with specific tree species. Specimens were collected in the summer of 2022. Only significant (*p* < 0.05) habitat associations are presented. The significance of IndVal indices was assessed using 10,000 Monte Carlo permutations.

Tree Species	Species	IndVal	*p*	Freq
Beech	*Araneus marmoreus*	0.83	0.001	5
Beech	*Florinda coccinea*	0.43	0.002	9
Black walnut	*Frontinella pyramitela*	0.33	0.001	36
Black walnut	*Argiope aurantia*	0.27	0.001	61
Black walnut	*Mangora maculata*	0.08	0.001	122
Black walnut	*Mecynogea lemniscata*	0.07	0.001	151
Boxelder	*Micrathena gracilis*	0.11	0.001	120
Boxelder	*Verrucosa arenata*	0.09	0.001	121
Deadwood	*Parasteatoda tabulata*	0.74	0.001	17
Deadwood	*Rabidosa rabida*	0.74	0.001	17
Deadwood	*Agelenopsis pennsylvanica*	0.17	0.016	45
Deadwood	*Leucauge venusta*	0.06	0.001	172
Green ash	*Metepeira labyrinthea*	1.00	0.001	3
Japanese honeysuckle	*Agelenopsis aperta*	0.51	0.001	35
Jumpseed and hornwort	*Leucage* sp.	0.14	0.001	91
Pawpaw	*Gea heptagon*	1.00	0.001	17
Pawpaw	*Araneus cavaticus*	0.71	0.001	23
Pawpaw	*Micrathena sagittata*	0.34	0.001	32
Chinese privet	*Eustala anastera*	0.97	0.001	31
Slippery elm	*Acantherpeira stellata*	1.00	0.001	12
Slippery elm	*Gasteracantha cancriformis*	1.00	0.001	12
Slippery elm	*Tetragnatha* sp.	0.53	0.001	20
Tulip poplar	*Cicurina arcuata*	1.00	0.001	5
Virginia creeper	*Melpomene* sp.	1.00	0.001	5
White oak	*Araneus* sp.	0.60	0.001	9
White oak	*Atypus affinis*	0.40	0.009	6

## Data Availability

The datasets analyzed for this study are all contained with the manuscript.
